# Conserved and Distinct Modes of CREB/ATF Transcription Factor Regulation by PP2A/B56γ and Genotoxic Stress

**DOI:** 10.1371/journal.pone.0012173

**Published:** 2010-08-13

**Authors:** Naval P. Shanware, Lihong Zhan, John A. Hutchinson, Sang Hwa Kim, Leah M. Williams, Randal S. Tibbetts

**Affiliations:** Department of Pharmacology, Program in Molecular and Cellular Pharmacology and Molecular and Environmental and Toxicology Center, University of Wisconsin School of Medicine and Public Health, Madison, Wisconsin, United States of America; Indiana University, United States of America

## Abstract

Activating transcription factor 1 (ATF1) and the closely related proteins CREB (cyclic AMP resonse element binding protein) and CREM (cyclic AMP response element modulator) constitute a subfamily of bZIP transcription factors that play critical roles in the regulation of cellular growth, metabolism, and survival. Previous studies demonstrated that CREB is phosphorylated on a cluster of conserved Ser residues, including Ser-111 and Ser-121, in response to DNA damage through the coordinated actions of the ataxia-telangiectasia-mutated (ATM) protein kinase and casein kinases 1 and 2 (CK1/2). Here, we show that DNA damage-induced phosphorylation by ATM is a general feature of CREB and ATF1. ATF1 harbors a conserved ATM/CK cluster that is constitutively and stoichiometrically phosphorylated by CK1 and CK2 in asynchronously growing cells. Exposure to DNA damage further induced ATF1 phosphorylation on Ser-51 by ATM in a manner that required prior phosphorylation of the upstream CK residues. Hyperphosphorylated ATF1 showed a 4-fold reduced affinity for CREB-binding protein. We further show that PP2A, in conjunction with its targeting subunit B56γ, antagonized ATM and CK1/2-dependent phosphorylation of CREB and ATF1 *in cellulo*. Finally, we show that CK sites in CREB are phosphorylated during cellular growth and that phosphorylation of these residues reduces the threshold of DNA damage required for ATM-dependent phosphorylation of the inhibitory Ser-121 residue. These studies define overlapping and distinct modes of CREB and ATF1 regulation by phosphorylation that may ensure concerted changes in gene expression mediated by these factors.

## Introduction

Members of the CREB/ATF subfamily of bZIP transcription factors, including CREB, CREM, and ATF1 were among the first stimulus-induced transcription factors to be identified. The seminal member of this family, CREB, was identified some twenty years ago as the major nuclear binding protein of the somatostatin cyclic AMP response element (CRE), an octanucleotide palindrome (TGACGTCA) that has been identified and functionally validated in several thousand mammalian genes [1], [2], [3]. CREB is now widely recognized as a critical regulator of gene expression, particularly in neuronal and metabolic contexts [4], [5]. In the nervous system, CREB contributes to long-term potentiation, memory fear conditioning, circadian rhythm entrainment, and neuron survival [5]. CREB is also an important regulator of glucose homeostasis and metabolic rate in mammals [4]. CREB is activated in diabetes, and hepatic or adipocytic CREB promotes hyperglycemia and insulin resistance [4], [6]. CREB has also been implicated as a proto-oncogene in acute myeloid leukemia, where it may promote cell survival and deregulation of the cell cycle [7], [8].

Activation of CREB occurs through phosphorylation of Ser-133 within the kinase-inducible domain (KID) (Reviewed in Ref. [1]). Canonical activation of CREB occurs in response to cAMP, which induces PKA-dependent Ser-133 phosphorylation. The phosphorylation of CREB on Ser-133 promotes recruitment of the histone acetyl transferase CREB-binding protein (CBP) and coactivation of CREB target genes harboring octanucleotide CRE sequences or variants thereof [9], [10]. CREB activation is also strongly dependent on the recruitment of CREB-regulated transcription co-activator (CRTC) proteins, which bind to the carboxyl-terminal bZip domain [11], [12]. CRTC-dependent activation of CREB does not absolutely require phosphorylation of Ser-133; however, CRTC binding to CREB facilitates phospho-Ser-133-dependent CBP recruitment and transcriptional activation [13]. Like CREB, CRTC proteins are essential regulators of metabolism in mammals [14].

ATF1 and CREM are structurally and functionally related to CREB and mediate cyclic AMP responses as homodimers or as heterodimers with CREB [1]. ATF1 and CREM display high amino acid identity to CREB particularly within the KID and bZIP regions and are phosphorylated and activated by PKA on conserved sites analogous to Ser-133 in CREB [1], [15]. Whereas CREB and ATF1 are expressed throughout development, and in most if not all tissues in the adult, the expression of CREM occurs in a more tissue-restricted fashion, with highest expression observed in testis [16]. CREB-deficient mice die perinatally from respiratory insufficiency while *ATF1*-null mice are phenotypically normal [17]. *ATF1* heterozygosity on a *CREB*-null background, however, causes early embryonic lethality [17]. Thus, CREB appears to be responsible for the bulk of essential functions carried out by CREB/ATF family members; however, the activities of ATF1 and CREM are not fully redundant with those of CREB.

Previous work has demonstrated that, in addition to its regulation by metabolic and growth signals, CREB is a target of the DNA damage response [18], [19], [20]. CREB is directly phosphorylated by the ATM protein kinase on Ser-111 in response to ionizing radiation (IR) and other types of genotoxic stimuli [18], [20]. The phosphorylation of CREB on Ser-111 primes phosphorylation of Ser-108, Ser-114, and Ser-117 by CK1 and CK2, which is required for additional ATM-dependent phosphorylation of Ser-121 [19]. All told, ATM, CK1, and CK2 phosphorylate five sites within the KID in response to DNA damage [19]. A net result of ATM/CK cluster phosphorylation is a decrease in the binding affinity between the KID domain of CREB and the KID-interacting (KIX) domain of CBP [19], [20]. However, although the ATM/CK cluster is positionally conserved in *Drosophila* CREB orthologs, its physiologic functions have not been elucidated. It is also unclear whether DNA damage-dependent phosphorylation is unique to CREB or represents a general mechanism of CREB/ATF regulation.

In this study we compared phosphorylation mechanisms of CREB and ATF1 both in the absence and presence of DNA damage. We show that ATM phosphorylates ATF1 in response to DNA damage on Ser-51, which is analogous to the Ser-121 phosphorylation site in CREB that inhibits CBP binding, and that the PP2A/B56γ phosphatase complex antagonizes DNA damage-induced phosphorylation of both proteins. Although these aspects of CREB and ATF1 phosphorylation are shared, the mechanisms and extent of DNA damage-independent phosphorylation of CK residues is divergent. We show that DNA damage-independent phosphorylation of CREB is induced during cellular growth and reduces the threshold of DNA damage required for subsequent IR-induced phosphorylation by ATM. Our findings thus provide greater insights into CREB/ATF1 regulation and suggest that DNA damage signaling input into these structurally related proteins is evolutionarily conserved.

## Results

### ATF1 is hyperphosphorylated in asynchronously growing cells

We have previously described a complex phosphorylation cascade involving interplay between ATM, CK1 and CK2 in the genotoxic stress-induced phosphorylation of CREB [19]. The end consequence of this cascade is the phosphorylation of five clustered.

**Figure 1:**
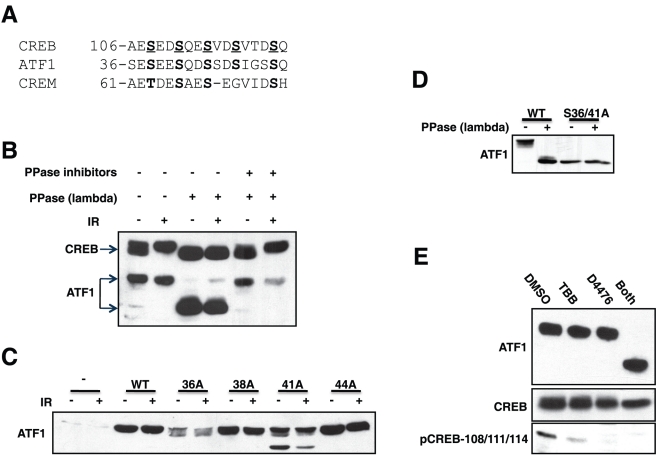
ATF1 is constitutively phosphorylated by CK1/CK2 *in vivo*. (**A**) Sequence overlay of ATM/CK cluster regions in CREB, CREM and ATF1. Homologous putative phosphorylation sites are shown in *boldface* and defined phosphorylation sites in CREB underlined. (**B**) ATF1 is basally phosphorylated in intact cells. HEK 293T cells were exposed to IR (10 Gy) or left untreated and cell extracts were prepared and treated with λ phosphatase (with or without inhibitors) prior to analysis by SDS-PAGE followed by immunoblotting with α-bZIP antibody that recognizes CREB and ATF1. The * denotes the position of a cross-reactive protein. (**C**) Phosphorylation site requirements for ATF1 electrophoretic mobility shift. HEK 293T cells were transfected with plasmids encoding Myc-ATF1^WT^ or the indicated Myc-ATF1 phosphorylation site mutants. Cell extracts were then made and analyzed by immunoblotting using α-Myc antibody. (**D**) The ATF1^S36/41A^ mimics *in vitro* dephosphorylated ATF1. HEK 293T cells were transfected with plasmids encoding Myc-ATF1^WT^ or the Myc-ATF1^S36/41A^ mutant. Cell extracts were prepared and treated with λ phosphatase prior to analysis by immunoblotting using α-Myc antibodies. (**E**) CK1 and CK2 inhibitors dephosphorylate ATF1. HEK 293T cells were treated with 75 µM D4476, 50 µM TBB or both compounds for 4 h. Cell extracts were then analyzed by immunoblotting using α-ATF1, α-CREB and α-pCREB-108/111/114 antibodies.

Ser residues: Ser-108, Ser-111, Ser-114, Ser-117, and Ser-121 (designated the ATM/CK cluster) within the amino-terminal region of the CREB KID. Although the functional consequences of CREB phosphorylation are not well understood, evidence suggested that ATM/CK cluster phosphorylation antagonized CREB-CBP interaction *in vitro* [19]. A sequence comparison of the KID region of the CREB, ATF1, and CREM shows strong positional conservation of the CK sites in both CREM and ATF1; however, only ATF1 showed co-conservation of the Ser-Gln dipeptide motifs in CREB that are phosphorylated by ATM *in vitro* and in intact cells (Fig. 1A and [21]). Based on this homology, we sought to test if ATF1 possessed a functional ATM/CK cluster that was a target of the DNA damage response.

CREB undergoes a rapid ATM dependent and phosphatase-sensitive electrophoretic mobility shift on SDS-PAGE gels following cellular exposure to IR [19], [20]. ATF1, however, migrated as a single band of 35 kDa on SDS-PAGE gels that was not affected by IR (Fig. 1B). Nevertheless, phosphatase treatment of cell extracts collapsed the major 35 kDa ATF1 band to a species with an approximate molecular mass of 27 kDa (Fig. 1B). This finding indicated that ATF1 was stoichiometrically hyperphosphorylated in asynchronously growing cells and that IR did not induce a detectable ATF1 electrophoretic mobility shift. Constitutive hyperphosphorylation of ATF1 was also observed in several other cell lines and primary mouse tissues from the spleen and thymus (Fig. S1A).

### CK sites regulate ATF1 hyperphosphorylation

We tested whether ATF1 hyperphosphorylation required the conserved CK sites. Of four candidate sites mutated to Ala (Ser-36, Ser-38, Ser-41, and Ser-44), Ser-36 and Ser-41 caused a change in ATF1 electrophoretic mobility; a fraction of each mutant migrated faster than the wild-type protein (Fig. 1C). Combined mutation of Ser-36 and Ser-41, in the ATF1^S36/41A^ mutant totally abolished the ATF1 electrophoretic mobility shift (Fig. 1D). This finding suggested that Ser-36 and Ser-41 within the putative ATM/CK cluster were phosphorylated in HEK 293T cells.

To demonstrate site-specific phosphorylation of ATF1 *in vivo*, we attempted and failed to generate a phospho-specific antibody that would recognize ATF1 phosphorylated on Ser-36, Ser-38, and Ser-41. As an alternative approach, we exploited the strong conservation between CREB and ATF1 in the KID region and constructed an ATF1^D39E,E41D^ mutant that rendered the ATF1 ATM/CK cluster identical to its CREB counterpart, which was readily detected using an α-pCREB-108/111/114 CREB antibody (Fig. S1B). These data suggested that ATF1 is always hyperphosphorylated on the CK sites *in vivo*. Also, the antibody reactivity suggested that in addition to Ser-36 and Ser-41, Ser-38 and Ser-44 were phosphorylated *in vivo*.

The ATF1 KID harbors a Ser-X-X-Ser-X-X–Ser motif making it a strong candidate for sequential phosphorylation by CK1 and CK2 (Fig. 1A and [22], [23]). To test this idea, we treated HEK 293T cells with the CK1 inhibitor D4476 [24] or the CK2-specific inhibitor TBB [25] and examined ATF1 electrophoretic mobility. Treatment with D4476 or TBB alone had no effect on ATF1 electrophoretic mobility (Fig. 1E). However, combined treatment with both inhibitors caused ATF1 to migrate as the hypophosphorylated species on SDS-PAGE gels (Fig. 1E). These findings suggest that the activity of CK1 or CK2 is sufficient to maintain ATF1 in a hyperphosphorylated state in HEK 293T cells.

### DNA damage-induced phosphorylation of ATF1

**Figure 2:**
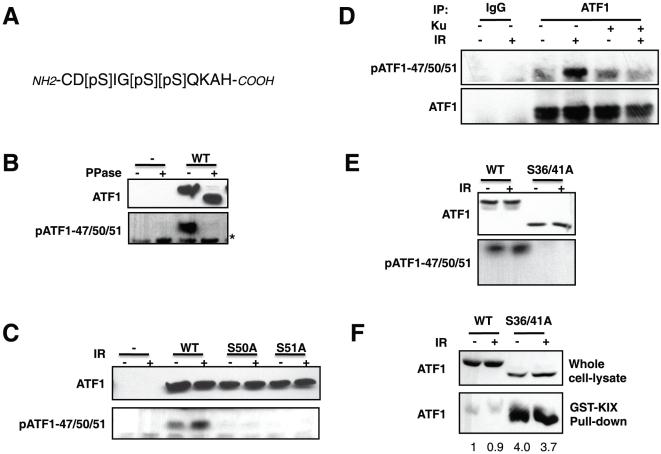
DNA damage induces ATM dependent phosphorylation of ATF1 on Ser-51. (**A**) Sequence of peptide antigen used to generate α-pATF1-47/50/51 antibody. (**B**) Phosphatase sensitivity of α-pATF1-47/50/51 antibody. HEK 293T cells were transfected with Myc-ATF1^WT^ plasmid. Cell extracts were prepared and treated with λ phosphatase prior to analysis by immunoblotting using α-Myc and α-pATF1-47/50/51 antibodies. (**C**) Phosphorylation site requirements and IR inducibility. HEK 293T cells were transfected with vector DNA (−), Myc-ATF1^WT^, Myc-ATF1^S50A^ or Myc-ATF1^S51A^ plasmids and exposed to IR (10 Gy, 2 h). Cell extracts were prepared and analyzed by immunoblotting using α-Myc and α-pATF1-47/50/51 antibodies. (**D**) IR dependent ATF1 phosphorylation is ATM dependent. HEK 293T cells either left untreated or treated with IR in the presence of 10 µM ATM inhibitor (KU-55933). Immunoprecipitation reactions were performed using a mock antibody or α-ATF1 antibody and immunoprecipitates were analyzed by immunoblotting using α-Myc and α-pATF1-47/50/51 antibodies. (**E**) The ATF1^S36/41A^ mutant is defective for IR induced pATF1-47/50/51 phosphorylation. HEK 293T cells were transfected with plasmid DNA encoding Myc-ATF1^WT^ or the Myc-ATF1^S36/41A^ mutant and either left untreated or subjected to 10 Gy IR for 2 h. Cell extracts were prepared and analyzed by immunoblotting using α-Myc and α-pATF1-47/50/51 antibodies. (**F**) Hyperphosphorylated ATF1 shows reduced binding to the KIX domain of CBP. HEK 293T cells were transfected with plasmids encoding Myc-ATF1^WT^ or the Myc-ATF1^S36/41A^ mutant and either left untreated or subjected to 10 Gy IR. Cell extracts were prepared 2 h later, incubated with GST-KIX-loaded beads, and bound protein analyzed by immunoblotting using α-Myc antibodies. Numbers under GST-KIX result demote fold changes in ATF1 levels.

While our previous data showed that ATF1 was stoichiometrically phosphorylated on CK sites *in vivo*, it was still not clear if ATF1 is a target of the DNA damage response. Ser-51 is positionally analogous to Ser-121 in CREB, which undergoes ATM-dependent phosphorylation in response to DNA damage (Fig. 1A). To test if Ser-51 is a target of the DNA damage *in vivo*, we generated an antibody against a peptide containing phosphorylated Ser-47, Ser-50 and Ser-51, using the rationale that Ser-47 and Ser-50 are likely phosphorylated by CK1 and CK2 (Fig. 2A). The α-pATF1-47/50/51 antibody was first tested against HEK 293T cell extract overexpressing Myc-tagged ATF1. The antibody displayed strong phosphatase-sensitive reactivity with Myc-ATF1 in HEK 293T cells (Fig. 2B). We further tested the specificity and IR-inducibility of the α-pATF1-47/50/51 antibody by assessing the effects of single Ser→Ala substitutions at Ser-50 and Ser-51 on basal and IR-induced immunoreactivity. Whereas wild-type Myc-ATF1 exhibited modest IR-induced phosphorylation, Myc-ATF1 harboring Ala substitutions at Ser-50- or Ser-51- failed to react with α-pATF1-47/50/51, indicating that the integrity of these sites was required for antibody recognition (Fig. 2C).

We next tested the ATM-dependence of IR-induced ATF1 phosphorylation. Preincubation of HEK 293T cells with the ATM inhibitor KU-55933 [26] completely blocked the IR-induced phosphorylation of ATF1 on Ser-47/50/51, establishing that the IR-induced phosphorylation of this motif required ATM (Fig. 2D). Finally, we tested whether ATF1 phosphorylation on Ser-47/50/51 required priming phosphorylation of the upstream CK sites. HEK 293T cells overexpressing either Myc-ATF1^WT^ or Myc-ATF1^S36/41A^ vectors were mock irradiated or exposed to IR. Immunoblotting analyses with the α-pATF1-47/50/51 antibody showed that the Myc-ATF1^S36/41A^ mutant was completely defective for Ser-47/50/51 phosphorylation (Fig. 2E). Thus ATF1 hyperphosphorylation on upstream CK residues is a prerequisite for DNA damage-induced phosphorylation of Ser-47/50/51.

### ATF1 hyperphosphorylation regulates the ATF1-KIX complex

DNA damage-induced phosphorylation of CREB antagonized its association with the CBP KIX domain [19], [20]. To test the effects of ATF1 hyperphosphorylation on its KIX binding activity, we transfected HEK 293T cells with Myc-ATF1^WT^ or Myc-ATF1^S36/41A^ vectors, exposed the cells to IR, and performed ATF1 pull-down assays using the GST-KIX fusion protein. ATF1^WT^-KIX binding was only marginally reduced on exposure to IR (Fig. 2F). The ATF1^S36/41A^ mutant showed greater than four-fold higher binding affinity for the KIX domain when compared to wild-type ATF1, and the ATF1^S36/41A^-KIX interaction was completely resistant to the effects of IR (Fig. 2F). These data suggest that ATF1 hyperphosphorylation negatively regulates CBP binding.

### B56γ-PP2A antagonizes CK and ATM-dependent CREB phosphorylation

**Figure 3:**
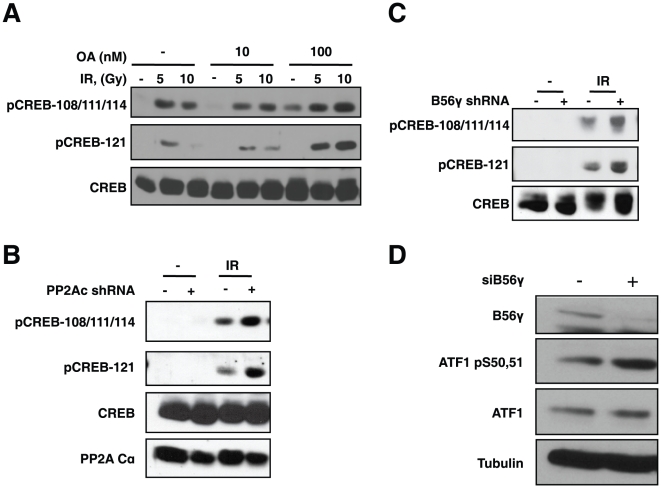
B56γ-PP2A mediates dephosphorylation of CREB and ATF1. (**A**) Okadaic acid (OA) sensitivity. HEK 293T cells were left untreated or exposed to 10 nM and 100 nM OA for 1 h. They were then subjected to IR for the indicated times. Cell extracts were prepared and analyzed by immunoblotting with α-CREB, α-pCREB-108/111/114 and α-pCREB-121 antibodies. (**B**) PP2Ac knockdown stimulates DNA damage-dependent CREB phosphorylation. HEK 293T cells expressing an shRNA targeting PP2Ac were compared to cells expressing a non-targeting shRNA construct. Cells were exposed to 10 Gy IR for 2 h and subjected to immunoblotting analysis with α-CREB, α-pCREB-108/111/114, α-pCREB-121 and α-PP2Ac antibodies. (**C**) B56γ knockdown stimulates DNA damage-dependent CREB phosphorylation. HEK 293T cells expressing an shRNA targeting B56γ were compared to cells expressing a non-targeting shRNA construct. Cells were exposed to 10 Gy IR for 1 h and subjected to immunoblotting analysis with α-CREB, α-pCREB-108/111/114, α-pCREB-121 and α-B56γ antibodies. (**D**) Effects of B56γ knockdown on ATF1 Ser-47/50/51 phosphorylation. HEK 293T cells were transiently transfected with control or B56γ siRNA and the levels of ATF1 Ser-47/50/51 phosphorylation assessed using α-pATF1-47/50/51 antibodies.

The above findings indicated that the vast preponderance of ATF1 molecules in intact cells are constitutively phosphorylated by CK1/CK2 within the ATM/CK cluster and that Ser-51 is a DNA damage-inducible site that is positionally analogous to the Ser-121 residue in CREB. To further explore the dynamic regulation of these residues, we sought to identify the relevant cellular phosphatases. Okadaic acid (OA) can be used to distinguish toxin-sensitive (PP1, PP2A and PP5) from toxin-insensitive (PP2B/calcineurin and PP7) phosphatases [27], [28]. Additionally, PP2A is extremely sensitive to OA inhibition and can be pharmacologically distinguished from less sensitive PP1 and PP5 [27]. As seen in Fig. 3A, both 10 nM OA treatment and 100 nM OA treatment led to elevated IR-induced CREB phosphorylation on both Ser-108/111/114 and Ser-121 in HEK 293T cells. In the absence of IR, 100 nM OA induced the phosphorylation of Ser-108/111/114, but not Ser-121, which is consistent with the idea that Ser-121 is strictly a DNA damage-inducible site. Neither overexpression of dominant-negative PP1 nor knockdown of PP5 affected basal or IR-induced CREB phosphorylation (data not shown). However, HEK 293T cells stably transfected with a shRNA construct directed against the PP2A catalytic subunit (PP2Ac) showed enhanced IR-induced phosphorylation on both Ser-108/111/114 and Ser-121, despite the partial nature of PP2A protein reduction in these cells (Fig. 3B). Thus, RNAi and inhibitor data suggested a role for the PP2A family of phosphatases in the regulation of CREB ATM/CK cluster dephosphorylation.

The PP2A catalytic subunit is targeted to diverse substrates via interaction with specificity-determining (B) subunits [29]. The B56γ B subunit has recently been implicated in the DNA damage response by regulating p53 phosphorylation [30], [31]. We evaluated the possibility that B56γ may be a CREB specific PP2A subunit by generating an HEK 293T cell line stably expressing a B56γ shRNA targeting all four B56γ isoforms. As seen in Fig. 3C, when compared to cells expressing control shRNA, IR-induced CREB phosphorylation on both Ser-108/111/114 and Ser-121 was elevated in B56γ shRNA-expressing cells. Transient transfection of 293T cells with B56γ siRNA also enhanced IR-induced phosphorylation of CREB phosphorylation on ATM/CK sites (data not shown). Finally, we found that B56γ knockdown also induced basal ATF1 phosphorylation on Ser-47/50/51 (Fig. 3D). These findings implicate B56γ-PP2A in the dephosphorylation of ATM/CK clusters in CREB and ATF1 in the presence and absence of DNA damage.

### DNA damage-independent phosphorylation of CREB by conditioned media (CM)

**Figure 4:**
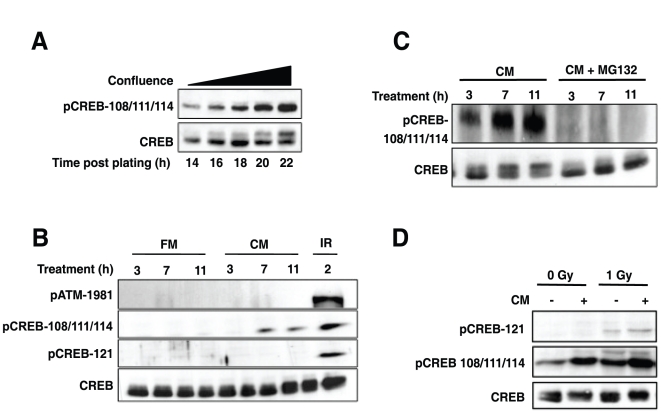
ATM and DNA damage-independent phosphorylation of the CREB ATM/CK cluster during cell growth. (**A**) CREB phosphorylation increases with time spent in culture. Replicate plates of HEK 293T cells were plated at 60–70% confluence and allowed to grow for 14 h. Cells were then harvested at 2 h intervals and the extracts analyzed by immunoblotting with α-CREB and α-pCREB-108/111/114 antibodies. (**B**) Conditioned media (CM) induces CREB phosphorylation independent of DNA damage. HEK 293T cells were plated overnight followed by exposure to fresh media (FM), or CM. A 10 Gy IR exposure (2 h) was used as a positive control to induce Ser-121 phosphorylation. Cell extracts were prepared at the indicated times and analyzed by immunoblotting with α-CREB, α-pCREB-108/111/114, α-pCREB-121, α-ATM and α- pATM-1981 antibodies. (**C**) MG-132 suppresses CM-induced CREB phosphorylation. HEK 293T cells were cultured in the presence of CM or CM supplemented with 10 µM MG132 (or solvent). Cell extracts were prepared at the indicated times after CM treatment and analyzed by immunoblotting with α-CREB and α-pCREB-108/111/114 antibodies. (**D**) CM-induced phosphorylation of CREB on Ser-111 facilitates IR-induced phosphorylation of Ser-121. HEK 293T cells were incubated with CM or not for 8 h and then mock irradiated or exposed to 1 Gy of IR. Cell extracts were analyzed by immunoblotting with α-CREB, α-pCREB-108/111/114 and α-pCREB-121 antibodies.

A major distinction between CREB and ATF1 ATM/CK cluster regulation pertains to the level of constitutive phosphorylation. While ATF1 is stoichiometrically phosphorylated on CK sites in the absence of DNA damage, only a fraction of CREB is phosphorylated on CK sites in undamaged cells (see Fig. 3 and Ref. [19]. During the course of cell growth experiments we noticed that the amount of constitutive Ser-108/111/114 phosphorylation on CREB in HEK 293T cells increased proportionally to the time spent in culture (compare lane 1 and lane 5 of CREB blot in Fig. 4A). This increase manifested as an increase in phospho-Ser-108/111/114 immunoreactivity and a characteristic CREB electrophoretic mobility shift as cells approached confluence. To further explore this phenomenon, we tested whether CM from HEK 293T cells grown to confluence was capable of inducing CREB phosphorylation. We found that CM, but not fresh medium, induced CREB phosphorylation on Ser-108/111/114 in freshly plated HEK 293T cells (Fig. 4B). CM had no effect on constitutive CREB Ser-121 phosphorylation or ATM Ser-1981 autophosphorylation (a measure of ATM activation status), which strongly implied the involvement of a DNA damage-independent pathway.

We used pharmacologic inhibitors to begin probing mechanisms DNA damage-independent CREB phosphorylation Neither PI3 kinase nor MEK inhibitors had any effect on CM-induced CREB phosphorylation in HEK 293T cells (data not shown), suggesting that these mitogenic pathways are not involved. On the other hand, preincubation of HEK 293T cells with the proteasome inhibitor MG-132 abolished CM induced CREB phosphorylation on Ser-108/111/114, suggesting a critical role for proteasome-mediated protein degradation (Fig. 4C). This finding is consistent with our previously published observations showing CREB phosphorylation was induced by the protein synthesis inhibitor, cycloheximide [32]. Although the pathways controlling CM-induced CREB phosphorylation are not clear, these findings suggest that the phosphorylation of these sites is dynamically regulated during cell growth in response to secreted factors.

Our previous studies showed that phosphorylation of CREB on Ser-108/111/114 was required for DNA damage-induced phosphorylation of CREB on Ser-121 [19]. It follows that increased phosphorylation of these residues in response to CM would potentiate DNA damage-induced phosphorylation of CREB on Ser-121 at low doses of IR. Consistent with this idea, we found that pretreatment of HEK 293T cells with CM for 6 h increased the phosphorylation of CREB in response to 1 Gy IR (Fig. 4D). These findings suggest that cellular growth status is a determinant of IR-induced CREB Ser-121 phosphorylation.

### CREB and ATF1 regulate ATM mRNA expression

**Figure 5:**
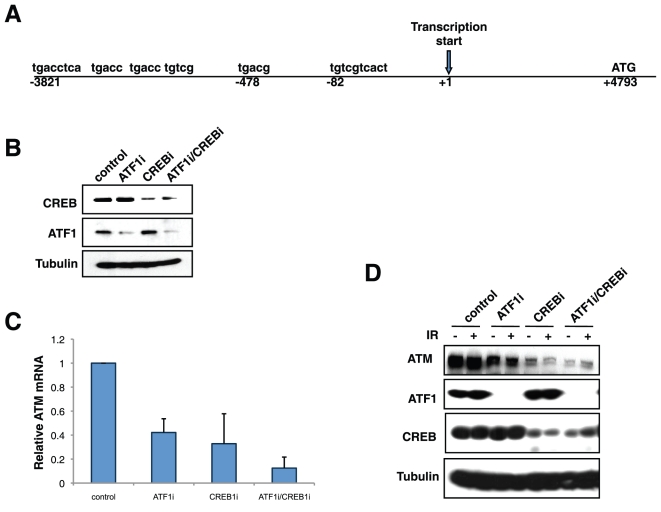
CREB/ATF1 regulate ATM transcription. (**A**) Schematic of the human ATM with putative CREs is shown. CREs conserved between human and mouse ATM promoters are in boldface. The arrow indicates the transcription start site. (**B**) Knockdown of CREB and ATF1 in MeWo cells. MeWo cells were transfected with shRNA for ATF1, siRNA for CREB or both and compared to cells expressing a non-targeting construct for 48 h. Cell extracts were subjected to immunoblotting with α-CREB, α-ATF1 and α-β-Tubulin antibodies. (**C**) Real-time PCR analysis of ATM mRNA in CREB/ATF1-deficient MeWo cells. Relative fold-expression levels of ATM mRNA normalized for GAPDH is shown. Error bars denote standard deviation from the mean from three independent experiments. (**D**) Effects of CREB and ATF1 knockdown on ATM protein levels. MeWo cells were transfected with shRNA for ATF1, siRNA for CREB or both and compared to cells expressing a non-targeting construct for 48 h before IR exposure. Cell extracts were prepared and subjected to immunoblotting with α-CREB, α-ATF1, α-ATM and α-β-Tubulin antibodies.

The ATM promoter contains positionally conserved CRE elements suggesting the interesting possibility that ATM is regulated by its own substrates (Fig. 5A and Ref. [2], [33]. To test if ATM is a direct target of CREB/ATF1 we transfected human MeWo melanoma cells with siRNA directed against CREB and ATF1 either individually or in combination. While ATF1 knockdown and CREB knockdown significantly reduced ATM mRNA levels, a double knockdown caused an almost 10-fold reduction in ATM mRNA levels (Figs. 5B and C). ATM protein levels were also significantly reduced in CREB/ATF1 knockdown cells (Fig. 5D). However, in no case did we observe effects of DNA damage on ATM mRNA or protein levels, indicating that although CREB and ATF1 contribute to constitutive ATM expression, it is unlikely that ATM-mediated phosphorylation of these factors modulates ATM promoter activity during DNA damage.

## Discussion

In this study, we compared the DNA damage-dependent and DNA damage-independent phosphorylation of CREB and ATF1 on an evolutionarily conserved ATM/CK phosphorylation cluster within their respective KIDs. The principle findings of the study include: (i) ATF1 is phosphorylated by ATM on Ser-51 in response to DNA damage in a manner that requires phosphorylation of upstream residues by CK1/CK2; (ii) ATF1 hyperphosphorylation on CK residues inhibits CBP binding; (iii) B56γ/PP2A antagonizes CREB and ATF1 phosphorylation on CK/ATM sites; and (iv) priming phosphorylation of CREB on CK sites during cell growth reduces the DNA damage threshold required for ATM-dependent phosphorylation of Ser-121. These findings illustrate conserved similarities as well as differences in the phosphoregulation of CREB and ATF1 and point toward the existence of a novel pathway regulating CREB phosphorylation status during cell growth.

CK-dependent phosphorylation of ATF1 within its KID, including Ser-36 and Ser-41, was reported by Masson et al, but the functions of these sites has been elusive [34]. Our studies show that virtually all ATF1 molecules are phosphorylated on the CK sites in asynchronously growing cells and that dual inhibition of CK1 and CK2 is required for their dephosphorylation (Fig. 1E). By contrast, positionally conserved CK sites in CREB are constitutively phosphorylated to a much smaller degree (Fig. 1E and Ref. [20]. Although the CK clusters are virtually identical between the two proteins (Fig. 1A), ATF1 contains three extra Ser residues and may be a preferred substrate for CK1 and CK2.

Although the functional impact of CK-mediated ATF1 phosphorylation is still unclear, we found that mutation of Ser-36 and Ser-41 increased CBP KIX domain binding by up to four fold (Fig. 2G). This result is consistent with the negative impact of CK-mediated phosphorylation on CBP binding affinity of CREB that we previously reported [20]. On the other hand, IR-induced phosphorylation of ATF1 on Ser-51 did not further reduce CBP-binding affinity (Fig. 2F), which contrasts with the inhibitory effects of IR-induced phosphorylation on CREB-CBP complex assembly [19]. To accommodate these findings, we propose that constitutive hyperphosphorylation by CK1/CK2 maintains ATF1 in an inactive state that promotes transcriptional repression. This conclusion is consistent with early studies showing that ATF1 is only weakly activated by cAMP [35] and more recent reports implicating ATF1 as a gene-specific repressor of transcription [36], [37]. An interesting question that emerges from this model concerns the impact of ATF1 hyperphosphorylation on the transcriptional activity of CREB/ATF1 heterodimers. It is possible that hyperphosphorylation of ATF1 confers an intermediate CBP-binding affinity to CREB/ATF1 heterodimers that allows for tuning of CREB-dependent transcriptional responses. In fact, CBP binding affinity is a primary determinant of CREB transcriptional activity [38].

This study implicates B56γ-PP2A as a negative regulator of ATM/CK cluster phosphorylation. Specifically, the IR-induced phosphorylation of CREB on both Ser-108/111/114 and Ser-121 was upregulated in PP2A- or B56γ-deficient cells, indicating that B56γ-PP2A extinguishes DNA damage-dependent phosphorylation of CREB (Fig. 3). CREB is the second DNA damage-regulated target for B56γ, with p53 being the other [30]. In the p53 paradigm, DNA damage-dependent phosphorylation of p53 on Ser-15 by ATM recruits B56γ-PP2A, which dephosphorylates the inhibitory phosphorylation site, Thr-55 [31]. By analogy, it is possible that DNA damage-dependent phosphorylation of the CREB ATM/CK cluster promotes B56γ-PP2A recruitment and dephosphorylation of proximal sites, such as Ser-133.

**Figure 6:**
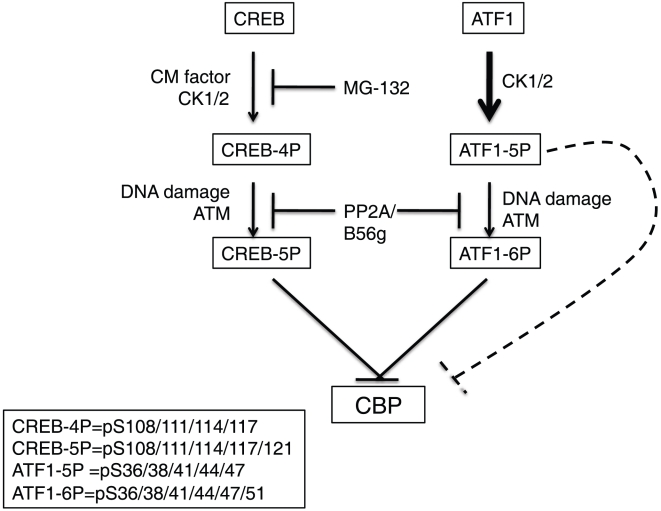
Working model depicting regulation of CREB and ATF1 on ATM/CK cluster residues. Both proteins are constitutively phosphorylated on CK residues resulting in CREB-4P and ATF1-5P isoforms that are further phosphorylated in response to DNA damage on Ser-121 and Ser-51, respectively to yield CREB-5P and ATF1-6P isoforms. Phosphorylation of CREB CK residues (Ser-108/111/114/117) is stimulated by a CM factor, whereas ATF1 CK residues (Ser-36/38/41/44/47) are constitutively phosphorylated (indicated by bold arrow). PP2A/B56γ antagonizes phosphorylation of ATM sites in both CREB and ATF1. Inhibition of CBP binding is one endpoint of ATM/CK cluster phosphorylation. The dashed line denotes that phosphorylation of CK sites in ATF1 is sufficient to inhibit CBP binding in the absence of DNA damage.

CREB differs from ATF1 with respect to the level of constitutive, DNA damage-independent, phosphorylation on the CK sites. Here, we show that the phosphorylation status of CREB CK residues is influenced by cell growth status and that CM from confluent cells induces Ser-108/111/114 phosphorylation in freshly plated cells (Fig. 4A and 4B). Furthermore, CM-induced phosphorylation of Ser-108/111/114 potentiated phosphorylation of CREB on Ser-121 in response to low dose IR, suggesting that the phosphorylation status of the CK residues determines the DNA damage threshold required for Ser-121 phosphorylation by ATM (Fig. 4D). Although the pathways governing DNA damage-independent CREB Ser-108/111/114 phosphorylation are not known, the proteasome inhibitor MG-132 effectively abolished the response, suggesting that degradation of a cellular factor is required (Fig. 4C). Although B56γ and/or PP2Ac are intriguing candidates, a 6 h treatment with MG-132 did not affect the expression of either (data not shown). Thus, an unidentified proteasome substrate apparently antagonizes DNA damage-independent CREB Ser-108/111/114 phosphorylation in cell lines. A model summarizing the ATF1 and CREB phosphorylation results is shown in Fig. 6.

The impact of DNA damage-dependent phosphorylation on CREB and ATF1 transcriptional activity is yet to be elucidated. The ATM gene harbors consensus CRE elements within its promoter that were required for optimal activity in reporter assays [33]. We showed that knockdown of CREB and ATF1 synergistically inhibited ATM mRNA expression, which supports functionality of the CRE elements (Fig. 5C). Although basal levels of ATM were reduced in CREB/ATF1-deficient cells, IR exposure did not induce or repress ATM in MeWO or HEK 293T cells, irrespective of CREB/ATF1 status (Fig. 5D and data not shown). These findings suggest that ATM does not regulate its own expression via phosphorylation of CREB and ATF1, but do not rule out this possibility. Additional studies using gene-targeted mice expressing phospho-mutant alleles of CREB should illuminate CREB-dependent transcriptional responses to DNA damage.

## Materials and Methods

### DNA constructs used

pCMV-Myc-hATF1 was constructed by cloning the BC029619 clone from Open Biosystems into the pCMV-Myc vector (Clontech). Site-directed mutagenesis was performed using the QuickChange method (Stratagene) and the indicated primers: ATF1^S36A^ (5′-CAACAGGTATCATCTTT AGCAGAAAGTGAGGAGTCC CAG-3′ and its reverse complement), ATF1^S38A^ (5′-GGTATCATCTTTATCAGAAGCTGAGGAGTC CAGGA-3′ and its reverse complement), ATF1^S41A^ (5′-GTCGGATGAGTCCTGGGCCTCCTCACT TTCTG-3′ and its reverse complement), ATF1^S36/41A^ (5′-CAGGTATCATCTTTAGCA GAAAGTGAGGAGGCCCAGGACTCATCC-3′ and its reverse complement), ATF1^S50A^ (5′-CTGACAGCATAGGCGCCTCACAGAAAGCTCAC-3′ and its reverse complement) and ATF1^S51A^ (5′-GACAGCATAGGCTCCGCACAGAAAGCT CACGGG -3′ and its reverse complement). The ATF1 shRNA construct was constructed by cloning the following oligonucleotide into the pSuperior plasmid: 5′-GATCCGAACTACACCTTCA-3′. The PP2Ac and B56γ shRNA constructs were constructed by cloning the following oligonucleotides into the pSuperior plasmid: PP2Ac: 5′-TGGAACTTGACGATACTC T-3′ and B56γ 5′-TCAGTGACAACGCAGCGAA-3′. siRNA SMARTpools against CREB, ATF1 and B56γ were obtained from Dharmacon Inc.

### Cell culture, antibodies and inhibitors

HEK 293T, MeWo, and HeLa cells were purchased through ATCC and maintained in Dulbecco's Modified Eagle's medium (DMEM) containing 5% FBS. The pATF1-47/50/51 antibody was generated by immunizing rabbits with a triply phosphorylated ATF1 peptide (NH2-CD[pS]IG[pS][pS]QKAH-COOH) (Cocalico Biologicals, Reamstown, PA). Peptide synthesis and purification of antisera was performed as described before for the pCREB-108/111/114 antibody[19]. Other antibodies used in this study include: α-Myc, α-CREB (bZip) and α-PP2Ac, and α-B56γ (SCBT); α-CREB, α-ATF1, and α-β-Tubulin (Millipore); α-ATM (Genetex); and α-pATM-1981 (Rockland). The CK1 inhibitor D4476 (4-(4-(2,3-Dihydrobenzo[1], [4] dioxin-6-yl)-5-pyridin-2-yl-1H-imidazol-2-yl)benzamide) and CK2 inhibitor TBB (4,5,6,7-Tetrabromo-benzotriazole) were purchased from EMD Biosciences and used at concentrations of 75 µM, 50 µM respectively. Okadaic acid (OA) was obtained from EMD Biosciences and added to culture media for 4 h at the indicated concentrations. Other reagents were from commercial sources and used as indicated. For Conditioned media (CM) generation, media from 3 million cells grown in 5 ml of DMEM with 10% FBS for 60 h was collected. The CM was treated for indicated lengths of time.

### Transfections and immunoblotting

Transfections were performed using the calcium phosphate DNA precipitation procedure as described. Cells were harvested 48 h later and extracts prepared as described previously [19]. Briefly, 75 µg of total protein was separated on 10% SDS-PAGE gels and transferred to Immobilon PVDF membranes (Millipore). Membranes were blocked in Tris-buffered saline containing 0.2% Tween-20 (TBS-T) and 5% dried milk and incubated overnight at 4°C with the indicated primary antibodies diluted in blocking solution. After washing, the blots were incubated with HRP-conjugated sheep anti-mouse or goat anti-rabbit secondary antibodies (Jackson) and developed using SuperSignal chemilluminescent substrate (Pierce). GST-KIX assays were performed as described previously [19]. Band pixel intensities for GST-KIX assay were determined using the density function of Quantity One software (Biorad).

### Real-time PCR analysis

Real-time PCR analysis of ATM mRNA was performed using standard procedures. Briefly RNA extraction was performed by the Qiagen RNA extraction kit followed by real-time PCR analysis using a Bio-Rad MyIQ single color real-time PCR detection system with SyBr green. The following ATM primers were used: 5′-CAAACGAACCTGGAGAGAGC-3′ and 5′- GGTGGAGGGATTTGGTAGG T -3′.

## Supporting Information

Figure S1 (A) ATF1 is hyperphosphorylated in mouse tissues. Thymus or spleen extracts were treated with vehicle or lambda phosphatase prior to analysis by immunoblotting with α-ATF1 and α-CREB antibodies. (B) ATF1E40D,D43E is detected by α-pCREB108/111/114 antibodies. HEK 293T cells were transfected with wild-type FLAG-ATF1 or FLAG-ATF1E40D,D43E expression plasmids. Overexpressed and endogenous ATF1 proteins were detected with α-ATF1 and α-pCREB108/111/114 antibodies. The detection of FLAG-ATF1E40D,D43E with α-pCREB108/111/114 provides evidence that the conserved ATM/CK cluster is phosphorylated in intact cells.
(1.82 MB EPS)
